# An investigation into how the European Working Time Directive has affected anaesthetic training

**DOI:** 10.1186/1472-6920-8-41

**Published:** 2008-08-12

**Authors:** Andrew R Bowhay

**Affiliations:** 1 Jackson Rees Department of Paediatric Anaesthesia, Royal Liverpool Children's Hospital, Alder Hey, Eaton Road, Liverpool, L12 2 AP, UK

## Abstract

**Background:**

The European Working Time Directive (EWTD) became law in 1993 but only applied to doctors in training in the United Kingdom in 2004. The trainees have in consequence had a reduction in their working hours but also a change to a shift pattern of working. For craft specialities, such as anaesthesia, there are concerns that a reduction in working hours has also led to a reduction in the time available for learning and that ultimately this may affect patient care. However, there is scant research on the perceptions of trainees concerning the impact of the EWTD on their training and working lives. This study investigated what the anaesthetic Specialist Registrars (SpRs) on the Mersey Deanery SpR rotation perceived to be training and also what effect the EWTD has had on that training and their quality of life, both within and outside work.

**Methods:**

The project was a cross sectional survey, using a quantitative questionnaire with qualitative free text comments which were aggregated into overarching themes and sub themes.

**Results:**

117 SpRs were sent questionnaires in April 2005; 73 completed questionnaires were returned (response rate 62.4%). Hierarchies of training opportunities emerged with training by consultants being most valued. 71.8% (95% CI 60.7 – 81.3) of trainees believed the EWTD has had a deleterious effect on their training and experience and 74.3% (95% CI 63.2 – 83.4) thought that they will be less prepared for a consultant post. 69.9% (95% CI 58.7 – 79.5) considered that their quality of life outside work had deteriorated, with only 15% (95% CI 8.3 – 24.6) finding improvement. 38.6% (95% CI 27.8 – 50.3) felt that they were not functioning as well as doctors, only 14.3% (95% CI 7.6 – 23.9) noting improvement. The trainees were still positive about anaesthesia and 73.2% (95% CI 62.2 – 82.5) would recommend this specialty to a student.

**Conclusion:**

The majority of anaesthetic SpRs in the Mersey Deanery have not welcomed the changes brought by the EWTD to their training, experience and quality of life outside work.

## Background

Long working hours have traditionally been a part of postgraduate medical training in the United Kingdom. The conventional work schedule of a long working day, of up to 12 hours, followed by a night on call and working the next day was not unusual and resulted in trainees working over 100 hours per week. However fatigue does affect performance[1,2]; for instance, speed and accuracy tests are significantly impaired after a night on call[3]. The effects are pronounced with moderate sleep deprivation of 17 to 19 hours having as deleterious effect on performance as a blood alcohol level of 0.05% and with longer periods without sleep, performance reaches levels equivalent to a blood alcohol level of 0.1%[4,5] and can lead to an increased risk of injuries to the trainee[6]. The concerns that tired doctors could have deleterious effects on patient safety[7,8], as well as on their own health, has led to pressure to reduce doctor's working hours.

In 1993 the European Working Time Directive (EWTD)[9] became law, however doctors in training were specifically excluded from its strictures until 2004. Subsequent revisions[10] and legal challenges[11,12] have now resulted in limitations of hours of work to an average of 58 hours per week (August 2004), 56 hours (August 2007) and 48 hours (2009). Prior to the EWTD, the on call pattern of working, for the majority of anaesthetic trainees in the Mersey Deanery, was 24 hours in the hospital; with the EWTD this has changed to a shift pattern[13], with restrictions to 13 hours work in a 24 hour period. There are concerns that the shift system is antipathetic to trainees' educational needs and the reduced hours of work have not led to job satisfaction[14,15]. A reduction in working hours does lead to a reduction in the time available for learning and gaining experience, particularly in the craft specialities, and the effects this may have on patient care is a concern for a number of specialities[16-19] including anaesthesia[20-22].

In 2002 the Conference of Postgraduate Medical Deans (COPMED) recognised that there would be educational implications of the EWTD and commissioned an ad hoc working group to look at its effects. Their report[23] stated that the EWTD would have a significant impact on the UK National Health Service (NHS), and that the nature and quality of training of the future medical workforce could be diminished. The report also challenges what is considered to be a learning or training opportunity for the trainee, who may compartmentalise the working day such that the time-honoured presumption that a business ward round or operating session cannot/does not involve teaching, and hence tend to differentiate between a business round and a teaching round. In light of this, it is therefore important to understand what trainees consider to be a training opportunity as this may differ from the views of their trainers.

There is scant information about perceptions of trainees concerning the impact of the EWTD[24]. This study addressed the views of trainees in one Deanery in the United Kingdom in the largest hospital based speciality (anaesthesia) on what they consider to be training opportunities as well as how the EWTD has affected their training, their lives outside work and how resultant deficiencies in training could be improved.

## Methods

Although undertaking a longitudinal cohort questionnaire survey, whilst the trainees were undertaking an on call rota and then resurveying them when they changed to a shift system was considered as a good method of assessing change, this was not feasible as some rotas had already changed and the trainee population was not stable, with trainees leaving and entering the rotation continually. Hence, this project was designed as a cross sectional survey, using a quantitative questionnaire with qualitative free text comments, which produced a "snapshot" of the anaesthetic Specialist Registrars (SpRs) perceptions of change since the EWTD came into force in one UK postgraduate Deanery. The questionnaire was developed and modified by presenting it to a group of 10 anaesthetic SpRs, selected by purposive sampling, who were asked to comment on the questionnaire to ensure that it is asked the appropriate questions clearly. By using purposive sampling it was possible to handpick trainees[25] to build up a sample which was culturally, age and gender diverse. It was then reviewed by a statistician and an expert in questionnaire design. The questionnaire was semi-structured using multiple choice and dichotomous questions for demographic data, and a Likert rating scale[26] for questions about perception of change, to assess the intensity views of the responses. In addition the awareness that questionnaires may not be completed carefully, wording can bias client's responses, they are impersonal and the full story may not be elicited led to a section being added for free text qualitative responses after each question. The qualitative free text comments from each respondent were aggregated for each question and overarching themes and sub themes were identified and coded for entry into the main database. The qualitative part of the study used the interpretive paradigm which began with the individual SpRs and set out to understand their multiple interpretations of, and perspectives on, the world around them; the emergent theory was grounded on the data generated by the research[27].

The questionnaire (see Additional file 1), with an accompanying explanatory letter and information sheet, were sent by post to each SpR at their work address for return by post from April 2005 to June 2005. All responses were anonymous. Follow up emails were sent after four and eight weeks to all SpRs, reminding them about the study and also giving them the opportunity to ask for another questionnaire if the original one had been misplaced. Each returned questionnaire was given a number, in sequence, as a unique identifier and the data were entered into an Access database.

Demographic data were quantified and analysed with non parametric statistical tests (Chi Squared test) with a P value of 0.05 or less than being considered significant.

All the SpRs were asked about their type of training number; a National Training Number (NTN) (EEA graduates) or a Visiting Training Number (VTN)(non-EEA graduates). The SpRs were also asked how long they had been on a shift rota. The shift rota became compulsory on 1 August 2004 so the minimum duration on this rota was 8 months. As a marker of how onerous their rota was, the trainee's were asked how many of them were on their tier of rota.

This research project received Local Research Ethical Committee (LREC) approval (REC reference number: 05/Q1502/11) as well as approval by the Research Committee of the Postgraduate Dean.

## Results

In April 2005, there were 122 SpRs on the Mersey Deanery database. Two were working overseas and three were on maternity leave. The remaining 117 SpRs were sent questionnaires; 73 completed questionnaires were returned (response rate of 62.4 percent).

The number of SpRs at each grade, their gender, training number type and achievement of the FRCA were similar for those who did not return the questionnaire (Table 1).

**Table 1:** The number of returned questionnaires for each SpR year, gender and training status and for the whole group.

	**Respondents**		**Total Group**	
	
**Grade, Gender and Training Status**	**Number in Grade**	**Percent**	**Number in Grade**	**Percent**
Locum Appointment (LAT)	4	5.5%	7	6.0%
SpR year 1	12	16.4%	22	18.8%
SpR year 2	17	23.3%	29	24.8%
SpR year 3	11	15.1%	23	19.7%
SpR year 4	13	17.8%	21	17.9%
SpR year 5	16	21.9%	15	12.8%
				
Female	29	42.0%	44	37.6%
Male	40	58.0%	73	62.4%
NTN	29	55.8%	64	58.2%
VTN	23	44.2%	46	41.8%
Possession of FRCA	50	68.5%	77	65.8%

Seventy percent of the SpRs achieved their first medical qualification between 1995 and 1999 (range 1982 to 2001).

Median time on a shift rota was 11 months (Interquartile range 8 to 13.5). Median number of SpRs on a rota was 8 (IQR 7 to 10). Four SpRs trainees (5.5%) reported that there were no on call rooms during their shifts. Catering facilities at night were available for 41/73 of the respondents.

SpRs were asked to summarise those aspects of their work which were considered to be a training opportunities using a Likert scale (Table 2). The majority of the examples were thought to be training opportunities, however a hierarchy did emerge. For instance, within the Intensive Care Unit (ICU) a teaching ward round had the most positive (87.7%) response compared to a handover ward round (46.4%). In the operating theatre working with a consultant during the day had the most positive responses (93.2%) followed by working with a consultant at night (85%), working on their own in theatre during the day (71.8%) and, least positive, working on their own in theatre at night (63.9%). Only 48.6% agreed that going on a pain ward round with a Pain Nurse Specialist was a training opportunity compared to 73.2% for attending a pain ward round with a consultant. Analysis of each part of this question showed no statistical difference between the responses for gender or type of training number. However, by aggregating the responses females gave more positive responses to the statements than males (374/427 (81.3%) vs 550/621 (72.6%), p = 0.001) and more SpRs with NTNs agreed with the statements compared to those with VTNs (262/444 (81.5%) vs 245/355 (69%), p < 0.0001).

**Table 2:** Results for Questions 1 with the statement and the number of responses to the statement and percentage response in brackets.

**Question Part**	Statement		**Response to Statement**				
		
**1**	The following are training opportunities.	**Number of Responses**	**Strongly Disagree**	**Disagree**	**Uncertain**	**Agree**	**Strongly Agree**
A	ICU teaching round	73	1 (1.4%)	5 (6.8%)	3 (4.1%)	32 (43.8%)	32 (43.8%)
B	ICU handover/business round	73	5 (6.8%)	21 (28.8%)	13 (17.8%)	27 (37.0%)	7 (9.6%)
C	Working on ICU at other times (Not A or B)	70	1 (1.4%)	12 (17.1%)	10 (14.3%)	39 (55.7%)	8 (11.4%)
D	Working with a consultant in theatre during the day	73	1 (1.4%)	1 (1.4%)	3 (4.1%)	34 (46.6%)	34 (46.6%)
E	Working with a consultant in theatre at night	73	0	5 (6.8%)	6 (8.2%)	40 (54.8%)	22 (30.1%)
F	Working on your own in theatre in the day	71	2 (2.8%)	6 (8.5%)	12 (16.9%)	42 (59.2%)	9 (12.7%)
G	Working on your own in theatre at night	72	4 (5.6%)	6 (8.3%)	16 (22.2%)	38 (52.8%)	8 (11.1%)
H	Working with a more senior trainee	72	1 (1.4%)	6 (8.3%)	5 (6.9%)	53 (73.6%)	7 (9.7%)
I	Working with a more junior trainee	73	1 (1.4%)	6 (8.2%)	14 (19.2%)	43 (58.9%)	9 (12.3%)
J	Going on a Pain ward round with a consultant	71	4 (5.6%)	7 (9.9%)	8 (11.3%)	38 (53.5%)	14 (19.7%)
K	Going on a Pain ward round with a Pain Nurse Specialist	72	6 (8.3%)	10 (13.9%)	21 (29.2%)	28 (38.9%)	7 (9.7%)
L	Attending Chronic Pain Clinics	71	0	8 (11.3%)	13 (18.3%)	34 (47.9%)	16 (22.5%)
M	Attending the FRCA course	69	1 (1.4%)	0	1 (1.4%)	27 (39.1%)	40 (58.0%)
N	Attending the Post fellowship SpR meetings	61	1 (1.6%)	1 (1.6%)	7 (11.5%)	33 (54.1%)	19 (31.1%)
O	Attending the local departmental educational meetings	73	0	4 (5.5%)	6 (8.2%)	42 (57.5%)	21 (28.8%)
P	Attending national meetings	69	1 (1.4%)	1 (1.4%)	1 (1.4%)	40 (58.0%)	26 (37.7%)

Subsequent questions asked about the impact of the EWTD on aspects of training and job satisfaction (Table 3). The majority of respondents 51/71 thought it had had a negative effect on training and 1/71 noted an improvement. The main themes that emerged from the free text comments for this question were that there was less teaching (24/59), more emergency work (12/59), they were at work more often (7/59) and they were getting less experience (7/59) (see Additional file 2 – Comment 1).

**Table 3:** Results for Questions 2 to 18 with the statement and the number of responses with percentage response in brackets.

Question Number	Question	Number of responses	Response to Question				
			
			Much Worse	Worse	No Change	Improved	Much Improved
2	How has the EWTD affected your training?	71	10 (14.1%)	41 (57.7%)	19 (26.8%)	1 (1.4%)	0
3	How onerous is the new rota, compared to the old one?	70	10 (14.3%)	35 (50.0%)	10 (14.3%)	14 (20.0%)	1 (1.4%)
4	How has the change in rota made attaining the competencies you require?	71	10 (14.1%)	36 (50.7%)	23 (32.4%)	2 (2.8%)	0
5	How has the change in rota affected your ability to attend departmental educational meetings?	71	16 (22.5%)	35 (49.3%)	18 (25.4%)	2 (2.8%)	0
6	If you are Pre FRCA – How has the new rota affected your ability to attend the FRCA course?	23	1 (4.3%)	9 (39.1%)	13 (56.5%)	0	0
7	How has the change in rota made obtaining annual and study leave?	71	6 (8.5%)	25 (35.2%)	38 (53.5%)	2 (2.8%)	0
			Much Less	Less	No Change	More	Much More
8	How has the new rota affected how much contact you have with consultants?	71	12 (16.9%)	32 (45.1%)	22 (31.0%)	3 (4.2%)	2 (2.8%)
9	How has the new rota affected the number of training opportunities you have?	68	9 (13.2%)	43 (63.2%)	12 (17.5%)	4 (5.9%)	0
10	How has the new rota affected the number of anaesthetics you give with a consultant?	71	12 (16.9%)	39 (54.9%)	12 (16.9%)	6 (8.5%)	2 (2.8%)
11	How has the new rota affected the number of anaesthetics you give on your own?	67	5 (7.5%)	22 (32.8%)	18 (26.9%)	20 (29.9%)	2 (3.0%)
12	How are you enjoying the new shift rota?	70	30 (42.9%)	18 (25.7%)	12 (17.1%)	10 (14.3%)	0
			Strongly Disagree	Disagree	Uncertain	Agree	Strongly Agree
13	Would recommend anaesthesia as a career to a medical student?	71	0	3 (4.2%)	16 (22.5%)	32 (45.1%)	20 (28.2%)
14	On call rooms should still be available at night even though you are doing full shifts.	73	0	1 (1.4%)	2 (2.7%)	10 (13.7%)	60 (82.2%)
15	Your training could be improved.	70	0	1 (1.4%)	17 (24.3%)	34 (48.6%)	18 (25.7%)
			Much Worse	Worse	No Change	Better	Much Better
16	How do you think the EWTD rotas are going to affect the readiness of SpRs to take up a consultant post after 5 years of SpR training?	70	8 (11.4%)	44 (62.9%)	18 (25.7%)	0	0
17	How has the new rota affected your quality of life outside work?	73	21 (28.8%)	30 (41.1%)	11 (15.1%)	9 (12.3%)	2 (2.7%)
18	How do you think the new rota has affected your functioning as a doctor?	70	7 (10.0%)	20 (28.6%)	33 (47.1%)	9 (12.9%)	1 (1.4%)

45/70 SpRs (question 3) felt that the new rota was worse or much worse than the old rota (see Additional file 2 – Comment 2); however 15/70 felt that it was an improvement (see Additional file 2 – Comment 3). The main themes that emerged were that the SpRs were more often at work (15/55), there was more emergency work (7/55), fatigue was worse (6/55) and that they felt "jet lagged" a lot of the time (5/55). However 5/55 SpRs felt that they were less fatigued with the new rota.

Half the SpRs (53.5%) felt that ease of obtaining study and annual leave had not changed (question 7)(see Additional file 2 – Comment 4), although 43.7% had noted a deterioration (see Additional file 2 – Comment 5).

Most SpRs (52/70) felt that their training could be improved (question 15). 9/48 SpRs felt that returning to the old style rotas and/or appointing more staff would improve training whilst 13/48 thought that it could be improved by better organisation and better rotas (12/48)(see Additional file 2 – Comment 6).

Although the SpRs reported that the number of anaesthetics they gave with a consultant had declined (question 10), there was no consensus on how the new rota has affected how many anaesthetics they give on their own (question 11), with 40.3% feeling that they are undertaking less, 32.8% more and 26.9% no change.

Overall the SpRs were unhappy with the new rota (question 12) with 68.6% thinking that it was worse or much worse and only 14.3% noting an improvement. 12/47 SpRs made references to the old 24 hour on call rota but there was divided opinion as to whether it was better or worse (see Additional file 2 – Comment 7). Of the positive responses to the question the main theme was an improvement in fatigue (see Additional file 2 – Comment 8).

The new rota has had a negative effect on the SpRs' quality of life outside work (Question 17) with 69.9% of SpRs stating that it was worse or much worse and only 15.1% noting an improvement. 28/50 SpRs noted a deleterious effect on family life (see Additional file 2 – Comment 9), with only 5 SpRs noting an improvement (see Additional file 2 – Comment 10).

The SpRs are concerned about the effect the new rota is having on their readiness to take up a consultant appointment at the end of their training (question 16) with 74.3% of the trainees considering that they will be less ready and none believing that they will be better prepared. The main reason (31/55) cited in the comments was that they would be less experienced.

The effect of the new rota on the SpRs' functioning as doctors (question 18) had a diverse response with 47.1% believing it had no effect (see Additional file 2 – Comment 11), 38.6% thought it was deleterious and only 14.3% thought there was an improvement. There were five main themes in the comments; less continuity of patient care (7/43), less experience (7/43), generally unhappiness with work (8/43), and increased tiredness (8/43), although a further 7/43 felt they were less fatigued.

The SpRs were very positive (73.2%) about recommending a career in anaesthesia to a medical student (question 13) with only 4.2% being negative (see Additional file 2 – Comment 12).

## Discussion

It is surprising that the anaesthetic SpRs have perceived the introduction of the EWTD in the Mersey Deanery as having a negative effect on their training as a reduction in working hours should in theory improve any sleep deprivation, with its deleterious consequences on performance[6,28], and their ability to learn[29,30]. Unfortunately the new shift rotas and the reduction of working hours did not coincide with an increase in staff, hence (although this study did not ask about precise rota patterns or sleep deprivation) a high proportion were undertaking rotas with the minimum number of staff for EWTD compliance (7 doctors per tier of rota) and would be undertaking a disproportionate amount of night work usually in blocks of 3 or 4 nights in a row. Although there is considerable variation between people, there is general agreement that shift work has a deleterious effect on sleep and has been likened to the effects of long distance travel[31]. The Mersey SpRs commented on feelings of fatigue, tiredness and "jetlag" and this has been reported by trainees in other specialities[32] undertaking shift work. As well as sleep disorders, shift work is recognised as having a deleterious effect on other aspects of health[31] and that females may be more effected than males[15] is concerning with their increasing proportion in the medical workforce. This study also confirms that hospitals have inadequate catering facilities at night[33] and staff frequently have to rely on snacks and deny themselves an adequate source of nutrition which can result in digestive disorders[34]. Whilst the main thrust of the EWTD was to improve the health and safety of doctors and patients, it is concerning that over a third of the Mersey SpRs believed the new rotas have had a deleterious effect on how they function as doctors, particularly with negative consequences on continuity of patient care. This is consistent with the Royal College of Physicians statement on the evidence it submitted to the House of Lords European Union Social and Consumer Affairs Sub-Committee G inquiry on the EWTD[35] in which they stated that

"We remain concerned that – in its present shape and form – compliance will have serious long-term effects for continuity of care, patient safety, and the education and training of doctors."

The experience gained by the Mersey SpRs has also been effected by the new rotas and although they were not asked to put a numerical figure on the number of anaesthetics they give, they do perceive that they are giving less anaesthetics overall which has been confirmed in other Deaneries[22,21]. The perception of gaining less experience, combined with difficulty in attaining the required competencies and workplace assessments, has led to a crisis of confidence; the SpRs know that, although they will be deemed to be competent at the end of their training, they will not have enough experience to be viewed as "experts" and hence will be less ready to cope with the challenges of a consultant appointment. The negative effects on training and experience of the EWTD have been recognised by the UK Post graduate Education and Training Board (PMETB)[36] who have noted that an undesired consequence has been the inevitable decrease in overall clinical exposure, which is crucial for developing experience and confidence. To compensate, they recommend that changes in the way education and training are undertaken are needed such that every working hour needs to contribute to the trainee doctor's education and practice to maximise their learning and training within compliant working patterns. The perception by the Mersey SpRs that they have much less contact and work less often with consultants emphasizes the importance of any contact being a learning experience; but they do put a different value on these training encounters as evidenced by the emergence of hierarchies of training opportunities for each training situation (e.g. Intensive Care Unit, Operating Theatre, pain ward round), with working with a consultant being viewed more positively than working solo at night. However, the value of working with a consultant is time dependent as working with one at night did not score as positively as working with one during the day; the Mersey SpRs realising that their supervising consultants are unlikely to be undertaking a EWTD shift rota and will have been working all day prior to their on call duties. This was succinctly summarised by respondent 34 "*....although the trainee may be fresh & eager to learn, the trainer may not be." *Hence a block of night shifts would be considered to have less training potential than a block of day time duties. Some training could be given by other shift workers who are not doctors[37], such as pain nurse specialists, however this is not as valued as that by consultants. Less contact with consultants has resulted in consultants having less confidence in the SpRs' abilities and are hence less likely to ask them to undertake complex anaesthetics and procedures on challenging patients[38]. A number of senior Mersey SpRs commented, like trainees in other specialities[16], that they would not wish to start training with the new shift rotas. The reduction in practical experience could be compensated by improvements in the formal educational programme, which the Mersey SpRs do value, however the EWTD has reduced their ability to attend these meetings, partly because the rigidity of the shift rota system makes it difficult to change or swap duties at short notice[39].

For the majority of Mersey SpRs, the deleterious effects on training of the EWTD have not been compensated by improvements in their quality of life outside work, particularly the effects on their families. A number noted that they were working more weekends and those with school age children felt they hardly ever saw them as when they were not working during the week, their children were at school. Mersey SpRs, whose partners were also doctors and working shifts, commented that it became very difficult for them to balance work and family life. It was not possible to ascertain from these data how many Mersey SpRs had medical graduate partners, however the Royal College of Physicians survey[40] found that 77% of the medical SpRs lived with a partner and 48% of these indicated that their partner was also a medical graduate and overall 44% of the SpRs had children.

Prior to the EWTD Aitken and Paice[41] found that 50% of anaesthetic trainees in one London region were in favour of shift working. This study has shown that trainees who have experienced shift working are less enthusiastic because they perceive that it has had a negative effect on their training (71.8%), enjoyment of work (68.6%) and quality of life outside work (69.9%), which is consistent with views from other specialities[39,40]. The Mersey SpRs were quite clear that one way of improving their training would be by increasing the number of trainees, or other grades, so that the rotas would be less onerous: A recommendation of 10 doctors on each tier of rota has been proposed[42]. Although the Department of Health aspires to have a future NHS led and delivered by consultants, this has not yet happened and consultant numbers are not sufficient to undertake the out of hours work of trainees.

The imposition of the new rotas has been viewed negatively by the group of Mersey SpRs however each individual trainee did have different opinions as to the cause of their dissatisfaction such as changes in their training, experience, work life balance or patient safety. However, in spite of that, 73.3% would still recommend anaesthesia as a career to a medical student – they accept that the EWTD affects all trainees whatever their speciality.

Limitations of the study are that the findings are limited to one Deanery and therefore cannot be generalised to the UK population of trainees, however some of the data do agree with the findings of studies in other specialities [39,40]. This cross sectional study does rely on the trainees' recall of their previous rotas which may change over time and although no data were available for sleep patterns, time at work or specific rota patterns, their subjective perceptions of the effects of change are important in a changing environment. A number of the questions asked about similar aspects of training in a different way (e.g. Questions 8 and 10) and produced very similar results implying that the trainees were answering the questions accurately and honestly.

## Conclusion

This study has shown what the anaesthetic SpRs in the Mersey Deanery consider to be training opportunities. They are unhappy with the changes the EWTD has brought because they perceive that it has affected their training and experience and that ultimately they will be less prepared for a consultant post than their predecessors. There has also been a deterioration in their quality of life outside work and that they are not functioning as well as doctors. Paradoxically, most trainees are still very positive about anaesthesia and would recommend this speciality to a student.

## Abbreviations

COPMED: Conference of Postgraduate Medical Deans; EEA: European Economic Area; FRCA: Fellowship of the Royal College of Anaesthetists; EWTD: European Working Time Directive; ICU: Intensive Care Unit; LREC: Local Research Ethics Committee; NHS: National Health Service; NTN: National Training Number; PMETB: Postgraduate Medical Education Training Board; RCA: Royal College of Anaesthetists; SpR: Specialist Registrar; VTN: Visiting Training Number.

## Competing interests

The author declares that they have no competing interests.

## Authors' contributions

AB conceived and designed the study, analysed and interpreted the data, drafted the article and approved the final version to be published.

## Pre-publication history

The pre-publication history for this paper can be accessed here:



## Supplementary Material

Additional File 1 EWTD Training Questionnaire. The questionnaire of the study completed by the Mersey SpRs. Click here for file

Additional File 2 Selected Comments of Respondents. The selected comments of respondents in the order they appear in the text. Click here for file
