# Supplementary material for: An investigation into how the European Working Time Directive has affected anaesthetic training
Source: BMC Med Educ. 2008 Aug 12;8:41. doi: 10.1186/1472-6920-8-41 (PMC2518917; doi:10.1186/1472-6920-8-41)
Supplement: Additional File 1 — EWTD Training Questionnaire. The questionnaire of the study completed by the Mersey SpRs. [file 1472-6920-8-41-S1.doc]

**EWTD Training Questionnaire**

Appendix A

Please complete the questionnaire and return to:

Andrew Bowhay

Consultant Paediatric Anaesthetist

Jackson Rees Department of Paediatric Anaesthesia

Royal Liverpool Children's Hospital

Alder Hey

Eaton Road

Liverpool

L12 2AP

**The questionnaire is anonymous.**

- **Present grade:** LAT  SpR1  SpR2  SpR3  SpR4  SpR5 
- **Status:** NTN  VTN  Pre FRCA  Post FRCA 
- **Gender:** M/F
- **Flexible:** Y/N

| **Year of Primary Medical Qualification** |  |
| --- | --- |
| **How long have you been on a full shift rota?** | months |
| **How many are there on your shift rota?** |  |
| **Is an on call room still available at night?** | Yes/No |
| **Is any catering available at night?** | Yes/No |

**Any other comments (please be candid):**

**Question 1**

| **1** | The following are training opportunities. | **Strongly**  **Disagree** | **Disagree** | **Uncertain** | **Agree** | **Strongly Agree** |
| --- | --- | --- | --- | --- | --- | --- |
| A | ICU teaching round |  |  |  |  |  |
| B | ICU handover/business round |  |  |  |  |  |
| C | Working on ICU at other times (Not A or B) |  |  |  |  |  |
| D | Working with a consultant in theatre during the day |  |  |  |  |  |
| E | Working with a consultant in theatre at night |  |  |  |  |  |
| F | Working on your own in theatre in the day |  |  |  |  |  |
| G | Working on your own in theatre at night |  |  |  |  |  |
| H | Working with a more senior trainee |  |  |  |  |  |
| I | Working with a more junior trainee |  |  |  |  |  |
| J | Going on a Pain ward round with a consultant |  |  |  |  |  |
| K | Going on a Pain ward round with a Pain Nurse Specialist |  |  |  |  |  |
| L | Attending Chronic Pain Clinics |  |  |  |  |  |
| M | Attending the FRCA course |  |  |  |  |  |
| N | Attending the Post fellowship SpR meetings |  |  |  |  |  |
| O | Attending the local departmental educational meetings |  |  |  |  |  |
| P | Attending national meetings |  |  |  |  |  |
|  | **Any other comments (please be candid):** | | | | | |

| Question 2 | **Much Worse** | **Worse** | **No Change** | **Improved** | **Much Improved** |
| --- | --- | --- | --- | --- | --- |
| How has the EWTD affected your training? |  |  |  |  |  |
| **Please qualify your answer here (please be candid):** | | | | | |
| Question 3 | **Much Worse** | **Worse** | **No Change** | **Improved** | **Much Improved** |
| How onerous is the new rota, compared to the old one? |  |  |  |  |  |
| **Please qualify your answer here (please be candid):**   | Question 4 | **Much Worse** | **Worse** | **No Change** | **Improved** | **Much Improved** | | --- | --- | --- | --- | --- | --- | | How has the change in rota made attaining the competencies you require? |  |  |  |  |  | | **Please qualify your answer here (please be candid):** | | | | | | | | | | | |

| Question 5 | **Much Worse** | **Worse** | **No Change** | **Improved** | **Much Improved** |
| --- | --- | --- | --- | --- | --- |
| How has the change in rota affected your ability to attend departmental educational meetings? |  |  |  |  |  |
| **Any other comments (please be candid):** | | | | | |
| Question 6 | **Much Worse** | **Worse** | **No Change** | **Improved** | **Much Improved** |
| If you are Pre FRCA – How has the new rota affected your ability to attend the FRCA course? |  |  |  |  |  |
| **Please qualify your answer here (please be candid):**   | Question 7 | **Much Worse** | **Worse** | **No Change** | **Improved** | **Much Improved** | | --- | --- | --- | --- | --- | --- | | How has the change in rota made obtaining annual and study leave? |  |  |  |  |  | | **Please qualify your answer here (please be candid):** | | | | | | | | | | | |

| Question 8 | **Much Less** | **Less** | **No Change** | **More** | **Much More** |
| --- | --- | --- | --- | --- | --- |
| How has the new rota affected how much contact you have with consultants? |  |  |  |  |  |
| **Please qualify your answer here (please be candid):** | | | | | |
| Question 9 | **Much Less** | **Less** | **No Change** | **More** | **Much More** |
| How has the new rota affected the number of training opportunities you have? |  |  |  |  |  |
| **Please qualify your answer here (please be candid):**   | Question 10 | **Much Less** | **Less** | **No Change** | **More** | **Much More** | | --- | --- | --- | --- | --- | --- | | How has the new rota affected the number of anaesthetics you give with a consultant? |  |  |  |  |  | | **Please qualify your answer here (please be candid):** | | | | | | | | | | | |

| Question 11 | **Much Less** | **Less** | **No Change** | **More** | **Much More** |
| --- | --- | --- | --- | --- | --- |
| How has the new rota affected the number of anaesthetics you give on your own? |  |  |  |  |  |
| **Please qualify your answer here (please be candid):** | | | | | |
| Question 12 | **Much Less** | **Less** | **No Change** | **More** | **Much More** |
| How are you enjoying the new shift rota? |  |  |  |  |  |
| **Please qualify your answer here (please be candid):**   | Question 13 | **Strongly**  **Disagree** | **Disagree** | **Uncertain** | **Agree** | **Strongly Agree** | | --- | --- | --- | --- | --- | --- | | Would recommend anaesthesia as a career to a medical student? |  |  |  |  |  | | **Please qualify your answer here (please be candid):** | | | | | | | | | | | |

| Question 14 | **Strongly**  **Disagree** | **Disagree** | **Uncertain** | **Agree** | **Strongly Agree** |
| --- | --- | --- | --- | --- | --- |
| On call rooms should still be available at night even though you are doing full shifts. |  |  |  |  |  |
| **Please give any reasons (please be candid):** | | | | | |
| Question 15 | **Strongly**  **Disagree** | **Disagree** | **Uncertain** | **Agree** | **Strongly Agree** |
| Your training could be improved. |  |  |  |  |  |
| **If you agree or strongly agree, please say how (please be candid):**   | Question 16 | **Much Worse** | **Worse** | **No Change** | **Better** | **Much Better** | | --- | --- | --- | --- | --- | --- | | How do you think the EWTD rotas are going to affect the readiness of SpRs to take up a consultant post after 5 years of SpR training? |  |  |  |  |  | | **Please qualify your answer here (please be candid):** | | | | | | | | | | | |

| Question 17 | **Much Worse** | **Worse** | **No Change** | **Better** | **Much Better** |
| --- | --- | --- | --- | --- | --- |
| How has the new rota affected your quality of life outside work? |  |  |  |  |  |
| **Please qualify your answer here (please be candid):** | | | | | |
| Question 18 | **Much Worse** | **Worse** | **No Change** | **Better** | **Much Better** |
| How do you think the new rota has affected your functioning as a doctor? |  |  |  |  |  |
| **Please qualify your answer here (please be candid):** | | | | | |
