# Supplementary material for: An investigation into how the European Working Time Directive has affected anaesthetic training
Source: BMC Med Educ. 2008 Aug 12;8:41. doi: 10.1186/1472-6920-8-41 (PMC2518917; doi:10.1186/1472-6920-8-41)
Supplement: Additional File 2 — Selected Comments of Respondents. The selected comments of respondents in the order they appear in the text. [file 1472-6920-8-41-S2.doc]

Additional File 1

Selected Comments of Respondents

Comment 1 - “Training time is reduced in theatres. Trainers & trainees need to use whatever time spent together to mutual benefit. For junior trainees hands on experience is reduced. On call time at night is not well utilised as although the trainee may be fresh & eager to learn, the trainer may not be.” (Respondent 34)

Comment 2 - “24 hr shift followed by a day off to recover/sleep, arrange affairs, study was viable. Current system involves repeated long shifts with no "wind-down" time. Recurrent cycle of work; drive home; eat; sleep; go to work. Leaves no time for social recreation/study/personal business. After 4 days, this is exhausting and demoralising. At day 4, I often feel unable to think clearly & this affects my competence.” (Respondent 20)

Comment 3 - “I prefer shifts, being a max of 12 hours, no matter how busy it is, you have sleep at the end! Although being a commuter (60 mile round trip) it means 2x as many journeys.” (Respondent 14)

Comment 4 - “If the leave is prebooked there are no probs. Emergency changes are that much more difficult as there tend to be sets of days/nights” (Respondent 44)

Comment 5 - “I organise rota. Hellish to give people what they want. Holidays ten d to have to fit in with the rota to remain compliant”. (Respondent 30)

Comment 6 - “Could be more focused, more "rigorous" (in a positive sense). E.g. Consultant and trainee can plan an accompanied list together, consultant delegates some cases to trainee, discusses them with him/her, expects trainee to have read up on them, gives feedback to trainee etc. Existing training opportunities could be used better. Some emergency work shifted to non-training grades.” (Respondent 3).

Comment 7 - “Working 24 hr duties previously was horrible. But now the more frequent duties (and reduced pay) is almost equally horrible, but probably safer for the patients (& doctors).” (Respondent 38)

Comment 8 - “Initially didn't like the concept of doing a lot of 12 hour shifts and losing 2 weekends in the process especially after the long days - going back home - all you do is sleep & come back the next day for work. But retrospectively - now it looks like probably it is less physically & mentally stressful when you don't work for 24 hrs continuously anymore.” (Respondent 40)

Comment 9 - “Hardly get to "see"/spend time with family. - Coincidently, if partner also in medical profession and both doing opposite shifts - don't get to see each other for a week to 10 days, living under one roof. - Domestic errands/ appointments remain pending for long.” (Respondent 59)

Comment 10 - “I feel I am spending more time with family especially with a 11 month child at home, it really helps working less hours in the hospital. No longer 24 hours - is psychologically better - quickly come home to your baby.” (Respondent 40)

Comment 11 - “Not sure - less tired and grumpy but less experienced than previous trainees probably and shift mentality has set in - i.e. - at least I won't have to do that case after 7pm etc, which I think is detrimental to patient care. - frequent handovers - all like Chinese whispers, no one really knows the patients anymore.” (Respondent 54)

Comment 12 - “Anaesthesia is one of the few specialities providing very good training by good approachable consultants. I do not think the shift system has anything to do with taking anaesthesia as a career because if the new rota affects training opportunities in anaesthesia, it must be having an equal or more adverse affect on other specialities as well.” (Respondent 23).
